# Gastroesophageal adenocarcinoma metastasizing to the chest wall: A case report and mini‑review of the literature

**DOI:** 10.3892/mi.2023.91

**Published:** 2023-06-14

**Authors:** Rebaz Ali, Omar H. Ghalib Hawramy, Fahmi H. Kakamad, Dlshad Hamasaeed, Soran H. Tahir, Deari A. Ismaeil, Bahra A. Awalmohammed, Hemn H. Kaka Ali, Bruj Jamil Mohammed, Hiwa O. Abdullah, Berun A. Abdalla

**Affiliations:** 1 Hiwa Cancer Hospital Centre, Sulaimani Directorate of Health, Sulaimani, Kurdistan 46000, Iraq; 2 Department of Scientific Affairs, Smart Health Tower, Sulaimani, Kurdistan 46000, Iraq; 3 College of Medicine, University of Sulaimani, Sulaimani, Kurdistan 46000, Iraq; 4 Kscien Organization for Scientific Research, Sulaimani, Kurdistan 46000, Iraq; 5 Department of Medicine, Shar Hospital, Sulaimani, Kurdistan 46000, Iraq

**Keywords:** esophageal cancer, chest wall, metastasis, adenocarcinoma, Ivor-Lewis esophagectomy, tumor dissemination

## Abstract

Subcutaneous metastasis from esophageal cancer (EC), particularly to the chest wall, is a very rare phenomenon. The present study describes a case of gastroesophageal adenocarcinoma that metastasized to the chest wall, invading the fourth anterior rib. A 70-year-old female presented with acute chest pain 4 months after undergoing Ivor-Lewis esophagectomy for gastroesophageal adenocarcinoma. A chest ultrasound revealed a solid hypoechoic mass on the right side of the chest. A contrast-enhanced computed tomography scan of the chest revealed a destructive mass on the right anterior fourth rib (7.5x5 cm). Fine needle aspiration revealed a metastatic moderately differentiated adenocarcinoma to the chest wall. Fluorodeoxyglucose (FDG)-positron emission tomography/computed tomography revealed a large FDG avid deposit on the right side of the chest wall. Under general anesthesia, a right-side anterior chest incision was made and the second, third and fourth ribs were resected with overlying soft tissues, including the pectoralis muscle and overlying skin. The histopathological examination confirmed a metastasized gastroesophageal adenocarcinoma to the chest wall. There are two common assumptions regarding chest wall metastasis from EC. The first one states that this metastasis can occur due to the implantation of the carcinoma during tumor resection. The latter supports the notion of tumor cell dissemination along the esophageal lymphatic and hematogenous systems. Chest wall metastasis from EC invading ribs is an extremely rare incident. However, its likelihood of occurrence should not be neglected following primary cancer treatment.

## Introduction

Esophageal cancer (EC) is the sixth major leading cause of cancer-related mortality. Almost half of the cases may already have metastasis at the time of presentation (1). It is mainly divided into two subtypes, including squamous cell carcinoma (SCC) and adenocarcinoma (ADC) (2). Esophageal ADC is associated with a poor survival rate and prognosis. However, at an early stage, its cure rate is ~50% following surgical resection (3,4). EC frequently spreads to the liver, lungs, bones and brain. However, subcutaneous and soft tissue metastases are uncommon (5,6). The incidence of subcutaneous metastasis originating from EC ranges from 0.7 to 9%, and only a few reports have been published to date (1,3,5-7). The risk factors for this type of metastasis remain ambiguous (3,7,8). The most common sites of subcutaneous metastasis from EC include the neck, scalp and face. Chest wall involvement is a very rare phenomenon (3). The majority of malignant tumors on the chest wall are metastasized from the cancers of surrounding organs (9).

The present study describes a rare case of gastroesophageal ADC that metastasized to the chest wall, invading the fourth anterior rib.

## Case report

### Patient information

A 70-year-old female presented to the Cardiothoracic Department at Smart Health Tower (Sulaimani, Iraq), complaining of acute chest pain after 4 months of undergoing an Ivor-Lewis esophagectomy for gastroesophageal ADC.

### Diagnostic assessment of the previous presentation

An endoscopic examination revealed an esophageal mass just above the gastroesophageal junction with evidence of bleeding. A computed tomography (CT) scan of the chest and abdomen revealed a lower esophageal tumor with local pathological lymph nodes without esophageal obstruction or other organ invasions. A biopsy specimen was obtained and histopathological examination (conducted at another institution) confirmed a gastroesophageal ADC. Immunohistochemistry was positive for CDX2 and negative for HER2 and TTF1 (data not shown; conducted at another institution). The patient received four cycles of neoadjuvant chemotherapy and then underwent an Ivor-Lewis surgery.

### Clinical findings and diagnostic assessment

The physical examination of the patient did not reveal any notable findings.

**Figure 1:**
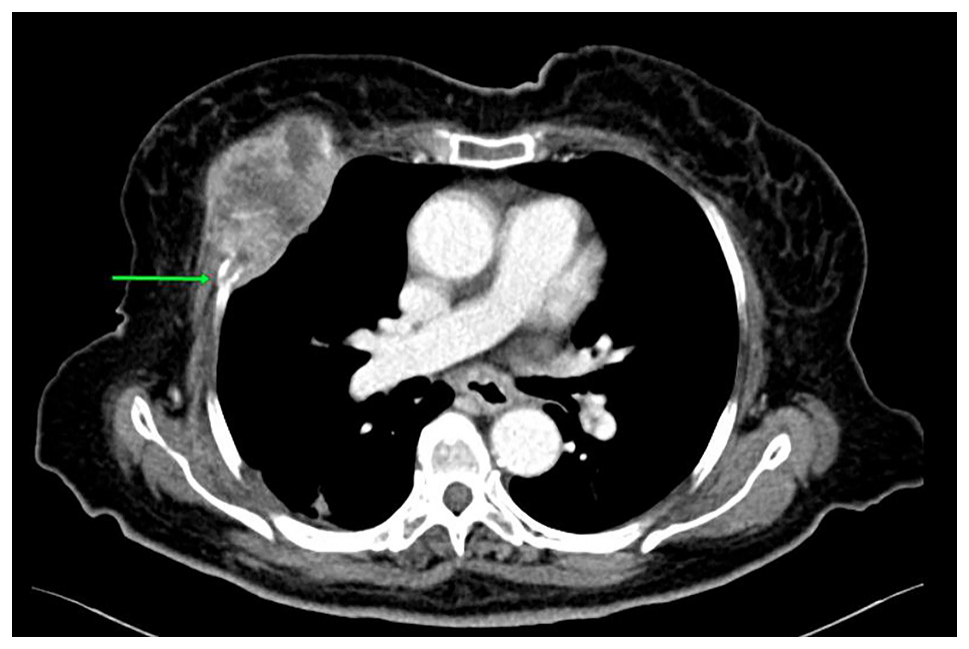
A contrast enhanced computed tomography scan of the chest, axial section. A heterogeneous enhanced soft tissue destructive mass on the anterior part of the right fourth rib (green arrow) was observed, consistent with bone metastasis.

At 4 months following the surgical resection of gastroesophageal ADC, the patient was referred to the Cardiothoracic Department at Smart Health Tower complaining of acute chest pain. A chest ultrasound (US) revealed a solid hypoechoic mass (87x63x51 mm) on the right side of the chest, lateral to the sternum, located ~12 mm under the skin. A contrast-enhanced CT scan of the chest revealed a destructive mass on the right anterior fourth rib (7.5x5 cm; Fig. 1). A fine needle aspiration (FNA) was conducted and this revealed a metastatic moderately differentiated ADC to the chest wall. A fluorodeoxyglucose (FDG)-positron emission tomography (PET)/CT scan revealed a large FDG avid deposit on the right side of the chest wall. The mediastinal lymph nodes exhibited internal calcification and were suspicious as infected lymph nodes. The lymph nodes exhibited a lack of uptake in the PET scan; they had calcification and were thus diagnosed as reactionary lymph nodes to the previous infection.

### Therapeutic intervention

**Figure 2:**
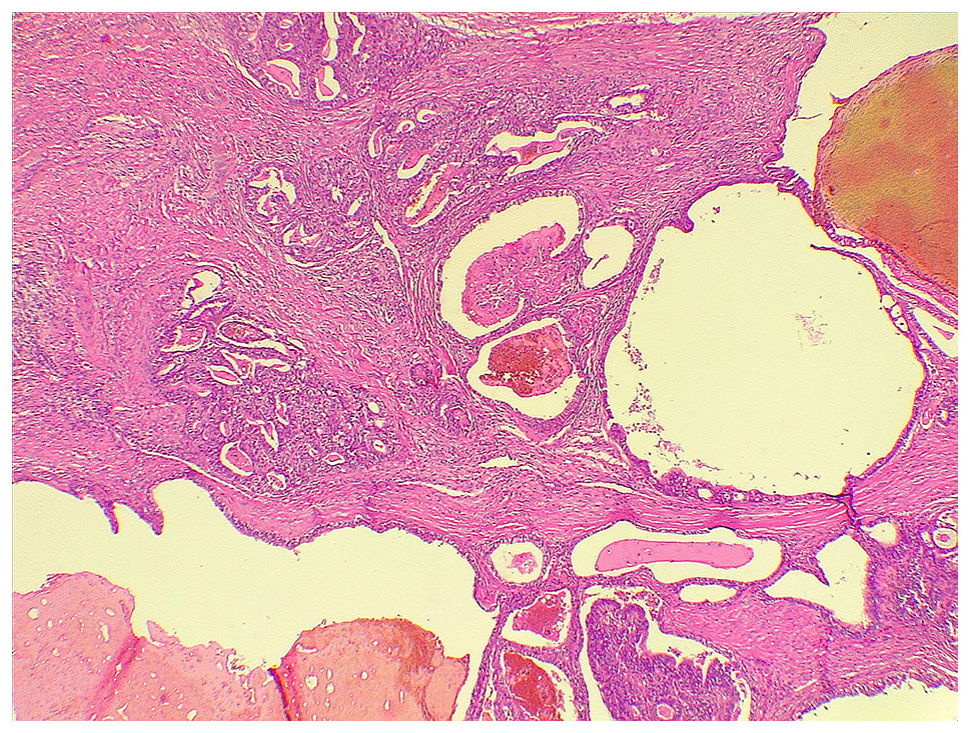
Histopathological analysis of the fibromuscular tissue revealed infiltration by malignant glands with areas of papillary formation associated with stromal desmoplastic reaction (magnification, x400).

The patient underwent a chest wall resection and reconstruction. Under general anesthesia, a right-side anterior chest incision was made and the second, third and fourth ribs were resected with overlying soft tissues, including the pectoralis muscle and overlying skin. A sample was sent for histopathological analysis and a gastroesophageal ADC metastasizing to the chest wall was confirmed (Fig. 2).

### Follow-up

The post-operative period was uneventful and the patient received Myogesic (six tablets) per day for 20 days (2x3).

## Discussion

EC is one of the most common causes of cancer-related mortality worldwide. The possibility of metastasis during the presentation of the primary cancer is up to 50%. Despite the fact that both types of EC (SCC and ADC) can respond to chemotherapy, the survival rate is low and is dependent on the disease stage. The 5-year survival rates of patients range between 95% at the early stages of the disease and 10-15% at stage III. The median survival rate in metastatic cases has been reported to be <1 year (1). This type of cancer predominately arises between the ages of 60 and 70 years (5). EC metastasis to visceral organs, such as the liver and lungs is common, while subcutaneous metastasis is estimated to be <10% (1,6,10). It is most likely asymptomatic, although it can also cause acute pain (11).

Although they are very rare, subcutaneous metastatic lesions can develop from both types of EC, and their incidence typically relates to the prevalence of the primary cancer. Esophageal ADC is more frequent among Caucasian males in contrast to esophageal SCC, which is more common among Caucasian females, Asians and individuals of African origin (3). Males have been mentioned as the dominant sex in cases of EC metastasis to unusual sites (2). In the esophagus, the most commonly involved regions are the lower and distal parts (12). In addition, it has been noted that tumors growing in the lower third of the esophagus, particularly ADC, have a higher risk of distant metastasis (16.7%) than those growing in the upper and middle third (~6%) (7). The case presented herein was a female in her 70s, who presented with acute chest pain following an Ivor Lewis esophagectomy for gastroesophageal ADC. In line with the findings of a previous study (7), the ADC developed in the lower part of the esophagus just above the gastroesophageal junction.

EC can metastasize to subcutaneous tissues of various locations and the most common ones are the neck, scalp and face. However, its metastasis to the back, axillary regions and chest wall is exceedingly uncommon (1,3). Datta *et al* (3) reported three cases of subcutaneous metastasis from esophageal ADC; two of them metastasized to the skin and scalp, and the third case involved the chest wall. They also stated that subcutaneous metastasis can occur in a variable time interval, such as in the early stage of the disease or later (3). Riley and Wah (13) also reported a case of skull and skin metastasis in a patient who experienced an esophageal ADC resection 4 years prior. The lesion was first suspected to be a post-traumatic hematoma (13). Another study discussed an incident of scalp metastasis from an esophageal ADC following a total esophagogastrectomy (14). In the current literature, at least to the best of our knowledge, the occurrence of chest wall metastasis from esophageal ADC has rarely been reported (13). Before Datta *et al* (3), Lindenmann *et al* (15) reported a chest wall metastasis from esophageal ADC in a 59-year-old male following 18 months of transhiatal esophagectomy and two-field lymphadenectomy. The case presented with thoracic pain and the tumor metastasized to the right ventrolateral area of the chest wall under the right pectoral muscle (15). Furthermore, Gogalniceanu *et al* (1) described a case of chest wall metastasis that appeared as a back lump that was noticed during the diagnosis of an esophageal ADC. Tunio *et al* (11) added another case to the literature that involved the right posterior chest wall and developed after 9 months of primary cancer management.

Generally, there are two common assumptions regarding chest wall metastasis from EC. The first one states that this metastasis can occur due to the implantation of the carcinoma during resection of the tumor and it is known as ‘implantation metastases’. The latter supports the notion of tumor cell dissemination along the esophageal lymphatic and hematogenous systems (15). In the case presented herein, chest wall metastasis was found 4 months following the surgical resection, in a manner that the condition presented with chest pain. The metastasis involved the right anterior of the chest wall, lateral to the sternum and invaded the anterior fourth rib, which is a rare finding. As regards the mechanism of metastasis, the case in the present study supports the second assumption and was considered to be caused by hematogenous dissemination.

Several particular points have been proposed for the improved diagnosis of chest wall metastasis. An extensive physical examination appears to be necessary in all patients who have been affected by a known cancer with repeated clinical assessments during the period of treatment and follow-up. In addition, multi-modality assessments need to be performed in patients with EC, such as a CT scan, PET scan, oesophago-gastro-duodenoscopy, endoscopic US, laparoscopy and biopsy of any chest wall lesion, in order to better consider the disease stage and prevent unnecessary intervention (1). Using US is essential to determine whether a lesion is solid or cystic, and it also demonstrates any relation of the lesion to surrounding structures (13). When a suspicious mass is identified on a US, CT and magnetic resonance imaging can be used as additional diagnostic approaches to provide further information about the nature of the lesion and its association with the adjacent structures (13). The combination of FDG PET and CT can accurately identify soft tissue masses originating in unusual locations (2). In the case of the present study, a chest US revealed a solid hypoechoic mass on the right side of the chest lateral to the sternum. A contrast-enhanced CT scan of the chest revealed a mass on the right anterior fourth rib. FNA revealed a metastatic ADC to the chest wall.

The survival rate of patients with subcutaneous metastatic tumors can be <1 year following diagnosis due to a poor prognosis. Commonly, the aim of treatment is palliation through tumor resection in addition to chemoradiotherapy (3). Systemic therapy may have a high failure rate in the treatment of such poor prognostic diseases, and Lindenmann *et al* (15) mentioned total resection of chest wall metastasis as the treatment of choice. The patient in the present study underwent chest wall resection and reconstruction, in which the second, third and fourth ribs were resected with overlying soft tissues. Histopathological analysis confirmed chest wall metastasis from previously managed gastroesophageal ADC.

In conclusion, chest wall metastasis from EC, invading the ribs is an extremely rare incident. However, its likelihood of occurrence should not be neglected, and proper clinical assessment and follow-up are mandatory following the treatment of primary cancer.

## Data Availability

The datasets used and/or analyzed during the current study are available from the corresponding author on reasonable request.
